# Thermal Behavior of Ti-64 Primary Material in Electron Beam Melting Process

**DOI:** 10.3390/ma14112853

**Published:** 2021-05-26

**Authors:** Jean-Pierre Bellot, Julien Jourdan, Jean-Sébastien Kroll-Rabotin, Thibault Quatravaux, Alain Jardy

**Affiliations:** Institut Jean Lamour—UMR CNRS 7198, LabEx DAMAS, Campus Artem, Université de Lorraine, 2 allée André Guinier, 54000 Nancy, France; julien.jourdan@univ-lorraine.fr (J.J.); jean-sebastien.kroll-rabotin@univ-lorraine.fr (J.-S.K.-R.); thibault.quatravaux@univ-lorraine.fr (T.Q.); alain.jardy@univ-lorraine.fr (A.J.)

**Keywords:** melting, electron beam, melting temperature, numerical simulation

## Abstract

The Electron Beam Melting (EBM) process has emerged as either an alternative or a complement to vacuum arc remelting of titanium alloys, since it is capable of enhancing the removal of exogenous inclusions by dissolution or sedimentation. The melting of the primary material is a first step of this continuous process, which has not been studied so far and is investigated experimentally and numerically in the present study. Experiments have been set up in a 100 kW laboratory furnace with the aim of analyzing the effect of melting rate on surface temperature of Ti-64 bars. It was found that melting rate is nearly proportional to the EB power while the overheating temperature remains roughly independent of the melting rate and equal to about 100 °C. The emissivity of molten Ti-64 was found to be 0.22 at an average temperature of about 1760 °C at the tip of the bar. In parallel, a mathematical model of the thermal behavior of the material during melting has been developed. The simulations revealed valuable results about the melting rate, global heat balance and thermal gradient throughout the bar, which agreed with the experimental values to a good extent. The modeling confirms that the overheating temperature of the tip of the material is nearly independent of the melting rate.

## 1. Introduction

Among the secondary remelting techniques, the EBM (Electron Beam Melting) process applies a high-power electron beam on the metallic material for melting, refining and controlling the casting and solidification stages. These operations ensure both the purification of the metal as it is gradually melted and the controlled solidification of the ingot in terms of structure and chemical homogeneity. 

**Figure 1:**
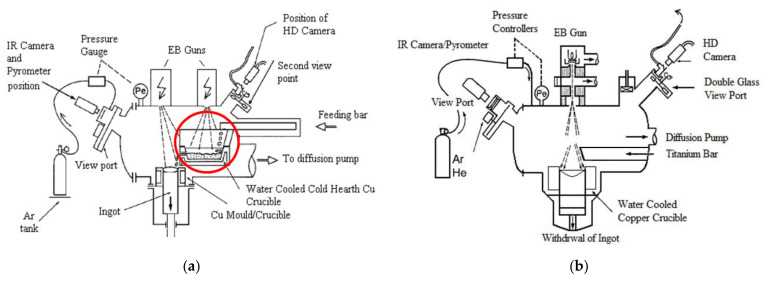
Schematic diagrams of: (**a**) EBCHM; (**b**) EBDM.

The EBM process can be used in two ways: Electron Beam Cold-Hearth Melting (EBCHM) and Electron Beam Drip Melting (EBDM). Schematic diagrams of these techniques are shown in Figure 1.

The main difference between these two methods is the inclusion of a cold hearth (circled in red in Figure 1a) in the EBCHM process as an intermediate refining stage between the melting and solidification steps. In the melting step, the electron beam heats and melts the bar tip. In the final solidification step, the molten metal is cast into a withdrawing water-cooled copper mold and solidified to form an ingot. All these operations are performed inside a vacuum chamber (10^−4^ to 10^−3^ mbar) in order to guarantee proper operation of the electron guns and to avoid alloy contamination. The EBM methods have a number of advantages when compared to the classic vacuum arc remelting (VAR) process, that lead to a better ingot quality. In particular, melting is conducted in a higher vacuum and for a longer time, thus enabling more complete degassing and dissolution of exogenous inclusions such as low-density inclusions known as hard-alpha [1,2].

The EBM processes are commonly used for manufacturing refractory or reactive metals, such as niobium and titanium [2,3]. Since these techniques essentially concern very high-performance materials used in leading-edge applications demanding exceptional reliability, it is extremely important to choose the most appropriate operating parameters in order to achieve optimum product quality. Despite experimental and numerical works having been performed on the refining and solidification steps [2,3,4,5], the thermal behavior of the raw material during the melting stage has only been investigated in the case of alternative processes such as vacuum arc remelting in the literature [6,7] although it plays an important role on the process operation. The surface temperature of the tip of the load activates the volatilization mechanism, which controls the material refining and the losses in alloying elements such as aluminum in Ti-64. Furthermore, the melting rate influences the liquid pool shape and depth, which have a direct effect on the ingot solidification structure. From a modeling point of view, it is obvious that the numerical simulations must include the melting stage of the process so that numerical predictions can be strictly correlated to operating conditions such as electrical parameters without the need to specify the melt rate value as an input data of the simulation. 

Thanks to the use of a well-dedicated electron beam laboratory furnace, a campaign of drip melting trials was performed with the aim of investigating the thermal behavior of a Ti-64 bar. Surface temperature of the bar tip was measured by pyrometer and infrared thermocamera, and the melting rate was recorded. As part of our modeling effort of the EBM processes, a mathematical model of the thermal behavior of the primary material (load) during melting has been developed. Details of this model are given in the present paper. The results of the model are compared to melt rate experimental data obtained with the laboratory EBM furnace, so that clear conclusions can be drawn.

## 2. Materials and Methods

### 2.1. Experimental Setup and Procedure

**Figure 2:**
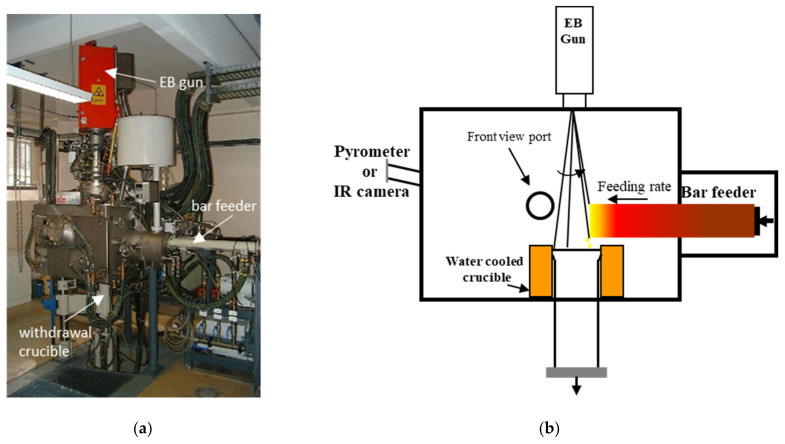
(**a**) Picture of the EB-Lab100 furnace; (**b**) schematic diagram of the Ti-64 bar melting.

A set of experiments was conducted in a 100 kW laboratory Electron Beam Melting furnace (ALD Vacuum Technologies—Lab100, Hanau, Germany) shown in Figure 2. The EB gun was used at an electric power from 11 kW to 25 kW with a constant beam voltage of 40 kV. The beam path is controlled by the Escosys pilot program (ALD Vacuum Tecnologies, Hanau, Germany), which allows automatic control of beam displacement, as well as a choice of beam pattern shape and frequency. A FLIR X6540SC infrared (IR) camera (Teledyne FLIR Systems, Thousand Oaks, CA, USA) with a wavelength of 2–5 μm was set up at one side of the furnace to evaluate the temperature of the bar tip as well as qualitative IR pictures of the bar side. This camera provides temperature measurements from low ambient temperature to beyond 2000 °C with an uncertainty of ±2% of measured values. The view port was equipped with a BaF_2_ glass transparent to infrared radiation. Since the glass view was the object of a possible quick coating with vaporized titanium and aluminum, a flow of argon in front of the viewpoint shutter was applied to prevent deposition. A two-wavelength pyrometer (IRCONE Modline Type-R, FLUKE Corp, Everett, WA, USA) was used to measure the surface temperature and to calibrate the infrared camera.

The Ti-64 bar (45 cm in length and with a section of 25.0 cm^2^) was placed in the bar feeder and the EB chamber vacuumed to below 5 × 10^−4^ mbar. The EB gun was then turned on, a first pattern being used to heat the bar tip while the ingot surface was heated by a second pattern. A few moments were spent in each trial on finding the exact required power of the beam for a steady melt according to the bar feeder speed. Initially, both the bar and ingot feeder speeds were changed manually; then, the speed of the ingot feeder was adjusted according to melting rate. Later, the bar and ingot feeder rates were set on automatic so that, when the quasi-stationary state is reached, the bar tip is maintained at the same location above the ingot crucible. During this quasi-stationary melting rate period temperature measurements using both pyrometer and IR camera were performed. After the consumption of 90% of the bar length, the beam was switched off and materials were cooled down. Finally, the post-experimental microstructure of the bar tips was observed and compared for different trials.

### 2.2. Numerical Modeling

This section describes the numerical modeling of the transient thermal behavior of the consumable Ti-64 bar in the EBDM process. An original feature of the model is the representation of liquid metal removal at the bar tip, which allows the model to predict the evolution of the melt rate with time, for given operating parameters.

#### 2.2.1. Heat Transfer Model

##### Model Equations

The modeling of the thermal behavior of the consumable bar is developed in a 2D axisymmetric geometry, enabling a representation of the curvature of the bar tip. Within the solid metal, heat is transferred by conduction. Under these conditions, the heat transport equation is written in the following form [8]:

(1) $${\rho C}_{p}\frac{\partial T}{\partial t}=\nabla \times \left(\lambda \vec{\nabla }T\right)$$

where T is the temperature, ρ the density, C_P_ the specific heat and λ the thermal conductivity. The temperature dependence of the alloy thermophysical properties is taken into account.

Dissipation of the latent heat of melting is represented using the equivalent specific heat method [9], which consists of replacing the specific heat in Equation (1) by

(2) $$C_{P}^{*}{=C}_{P}+\frac{\partial g_{l}}{\partial T}L$$

where L is the latent heat of melting, and g_l_ is the liquid fraction.

The calculation of the equivalent specific heat requires the knowledge of the solidification path, i.e., the evolution of the liquid fraction with temperature. In the case of a multicomponent alloy (such as Ti-64), the solidification path is in general unknown. Under these conditions and because the interval between liquidus and solidus is very small, we consider a linear variation of the liquid fraction between the solidus (T_sol_) and liquidus (T_liq_) temperatures. Such a variation implies the assumption of a uniform dissipation of the latent heat L in the temperature interval [T_sol_, T_liq_], which is particularly narrow in the case of Ti-64. Equation (2) can thus be written as

(3) $$C_{P}^{*}{=C}_{P}+\frac{L}{T_{\mathrm{liq}}{-T}_{\mathrm{sol}}}$$

Initially, the bar temperature is considered to be homogeneous, equal to the stub or pusher temperature (${T=T}_{0}$) i.e., the room temperature. The symmetry condition on the bar axis is given by ${\left(\frac{\partial T}{\partial r}\right)}_{r=0}\;=\;0$.

The boundary conditions on the edge and the tip of the bar depend on the process itself.

##### Boundary Conditions

At the bar tip, the kinetic energy of the electron beam is converted into thermal energy; however, the backscattering of electrons results in significant losses, which are in the range of 30% of the incident energy [10] for titanium. Thus, the heat power density applied to the tip surface of the bar is given by:

(4) $$\varphi_{\mathrm{EB}}=\frac{\varepsilon_{\mathrm{EB}\;}P_{\mathrm{EB}-\mathrm{tip}}}{{\pi R}^{2}}$$

where P_EB-tip_ is the electrical power of the beam applied to the tip, and ε_EB_ is equal to 0.7. In addition, the net heat exchanged by radiation between the liquid film and the furnace wall must be taken into account. Since the surface ratio between the bar and the furnace wall is very small and in the approximation of the grey body, the thermal flux density transferred can be expressed as:

(5) $$\varphi_{\mathrm{rad}}^{\mathrm{tip}}={\;\sigma \varepsilon }_{l}\left(T^{4}-T_{w}^{4}\right)$$

where T_w_ is the temperature of the furnace wall. 

At the bar lateral surface, heat transfer is controlled by thermal radiation between the material and the furnace walls with a similar expression as Equation (5):

(6) $$\varphi_{\mathrm{rad}}^{\mathrm{side}}={\;\sigma \varepsilon }_{s}\left(T^{4}-T_{w}^{4}\right)$$

At the end of the bar, a contact resistance between the stub or pusher and the bar is taken into account using a heat transfer coefficient h_sp_. This coefficient is assigned a constant value of 500 W.m^−2^·K^−1^, describing a moderately effective thermal contact between the pusher and the bar [11,12].

(7) $$\varphi_{\mathrm{push}}=h_{\mathrm{sp}}\left(T-T_{\mathrm{push}}\right)$$

The integration of these flux densities (Equations (4)–(7)) over the respective surface leads to heat power defined as $\varepsilon_{\mathrm{EB}}P_{\mathrm{EB}-\mathrm{tip}}$, ${\dot{Q}}_{\mathrm{rad}}^{\mathrm{tip}}$, ${\dot{Q}}_{\mathrm{rad}}^{\mathrm{side}}$, ${\dot{Q}}_{\mathrm{push}}$ for respectively the effective EB heat power, heat lost by radiation at the tip, heat lost by radiation at the bar side and heat lost at the pusher contact.

The thermal properties of Ti-64 used in the model are reported in Table 1. Note that the thermal emissivity of the liquid titanium has been obtained by IR and pyrometer measurements (see Section 3.2).

#### 2.2.2. Calculation Procedure

The heat transfer equation is solved using a finite volume method [15]. The numerical program called ‘Ebmelting’ is written in FORTRAN. A typical 160 × 1500 (r,z) orthogonal grid is applied for the spatial discretization. Furthermore, a fully implicit scheme is used for time discretization. During each time step (typically 0.5 s), we first calculate the temperature field in the material. Then, in order to simulate consumption of the bar associated to the fall of liquid metal droplets formed at the bar tip, the mesh cells whose temperature is greater than an “overheating temperature” T_oh_ are removed from the computational domain. After mesh cell removal, the boundary conditions are set at the new bar tip for the next time step. Calculations are performed until full consumption of the bar or until a given processing time. Note that the overheating temperature T_oh_ is an input variable for Ebmelting and is defined as the sum of the alloy liquidus temperature and a superheat.

Finally, Ebmelting calculates at each time increment the temperature field of the bar, the shape of the bar tip as well as the melting rate.

## 3. Results and Discussion

### 3.1. Experimental Melting Rate and Overheating Temperature

A set of eight trials has been set up in our laboratory electron beam furnace with associated melting rate and EB power as reported in Table 2. Notice that the total EB power P_EB-tot_ has two contributions, one is the electrical power used to scan the pattern at the bar tip P_EB-tip_ and the second is the power P_EB-ing_ applied to the ingot top for solidification control as schematically described in Figure 2b.

P_EB-tot_ = P_EB-tip_ + P_EB-ing_ (8)

**Figure 3:**
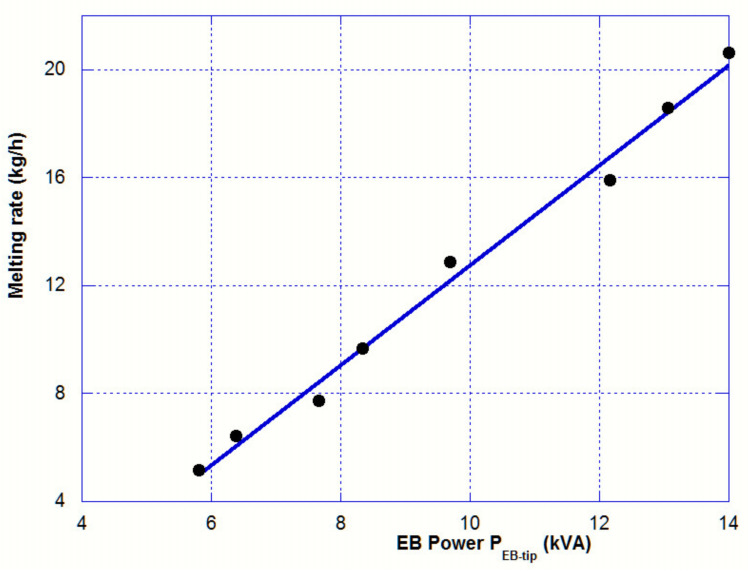
EB power P_EB-tip_ vs. melting rate. Slope = 1.8 kg/hr/kW.

Table 2 clearly reveals that the temperature of the bar tip is not correlated with the EB power applied, and its value remains in a range between 100 and 125 °C above the liquidus temperature. On the contrary, the melting rate increases almost linearly with the heating power as it is shown in Figure 3.

**Figure 4:**
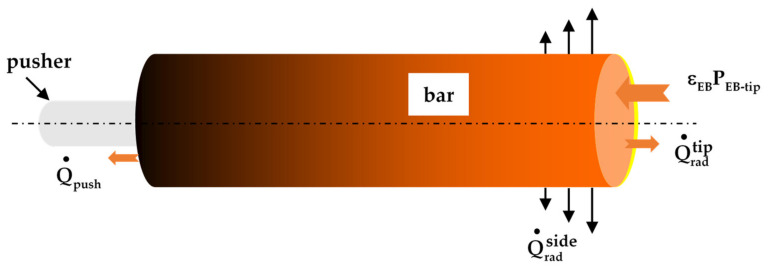
Heat fluxes at the bar boundaries.

During the quasi-stationary state, a straightforward heat balance based on the schematic Figure 4 leads to

(9) $$\left(h_{\mathrm{liq}}\left(T_{\mathrm{oh}}\right)-h_{\mathrm{sol}}\left(T_{0}\right)\right)\dot{m_{m}}=\varepsilon_{\mathrm{EB}}P_{\mathrm{EB}-\mathrm{tip}}-\left[{\dot{Q}}_{\mathrm{rad}}^{\mathrm{tip}}+{\dot{Q}}_{\mathrm{rad}}^{\mathrm{side}}+{\dot{Q}}_{\mathrm{push}}\right]$$

where h_liq_ and h_sol_ are respectively the specific enthalpy of liquid and solid, and $\dot{m_{m}}$ is the melting rate. The thermal power lost by radiation on the bar tip remains at a constant value (since the overheating temperature T_oh_ does not change with the beam power), and the heat lost on the pusher side can be considered as negligible during the quasi-stationary regime.

If the heat lost by radiation on the bar side ${\dot{Q}}_{\mathrm{rad}}^{\mathrm{side}}$ was independent of the EB heat power applied on the bar tip, then the melting rate would be proportional to that EB power applied and the slope of the adjusted line would be equal to:

(10) $$S=\varepsilon_{\mathrm{EB}}{\left(h_{\mathrm{liq}}\left(T_{\mathrm{oh}}\right)-h_{\mathrm{sol}}\left(T_{0}\right)\right)}^{-1}$$

The analytical value of S (1.5 kg/hr/kW) does not match with the experimental slope (see Figure 3), which is equal to 1.8 kg/hr/kW. We will see later (Section 3.5) that the temperature profile on the bar side cannot be considered as independent of the beam power. Therefore, the assumption of an independent radiation heat flux on the bar side is wrong.

### 3.2. Emissivity Measurements and Temperature Profiles

In a first step, the temperature of the bar tip is continuously measured with the IR camera during a cooling test (EB is sharply switched off). The emissivity is then selected such as the measured temperature of the phase change ranges between 1650 °C (T_sol_) and 1670 °C (T_liq_). An emissivity of 0.22 was obtained. In a second step, the average surface temperature of the bar tip was monitored by the IR camera during a drip melting run and compared to the temperature measured with the pyrometer. Again, the value 0.22 was required to match IR and pyrometer temperatures. This value is in agreement with the findings of Choi [16] and Rai [17] (0.23 and 0.20, respectively).

**Figure 5:**
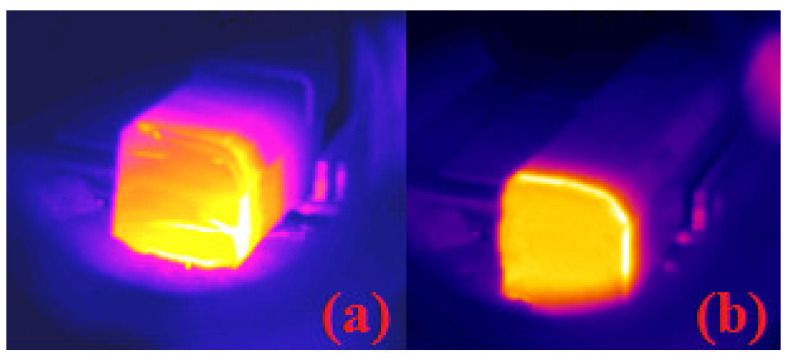
Temperature visualization for two conditions: (**a**) low melting rate—8 mm/min and (**b**) high melting rate—20 mm/min.

An example of IR picture is presented in Figure 5 for a low (Run#1) and a high (Run#5) melting rate. It clearly emphasizes the steeper temperature gradient at high melting rate. The heat supplied by the EB diffuses more deeply into the bar at lower melting rate.

### 3.3. Microstructure of the Bar Tip

In order to get a better understanding of the thermal treatment experienced by the bar during electron beam drip melting (EBDM), the microstructure of the remaining bar tips, after two heats performed at distinguishable melting rates, was studied. In both cases, the melt was sharply interrupted thanks to the EB switch-off. Two samples were obtained from the experiments with a bar feeding speed of 8 and 20 mm/s (melting rate of 5.2 and 12.9 kg/h, respectively). The samples were prepared by cutting in the middle longitudinal section the first tens of millimeters from the front surface of the bar, then polished and etched by Kroll agent. Finally, they were examined under Zeiss-Axioplan 2 optical microscope (Zeiss, Iena, Germany).

**Figure 6:**
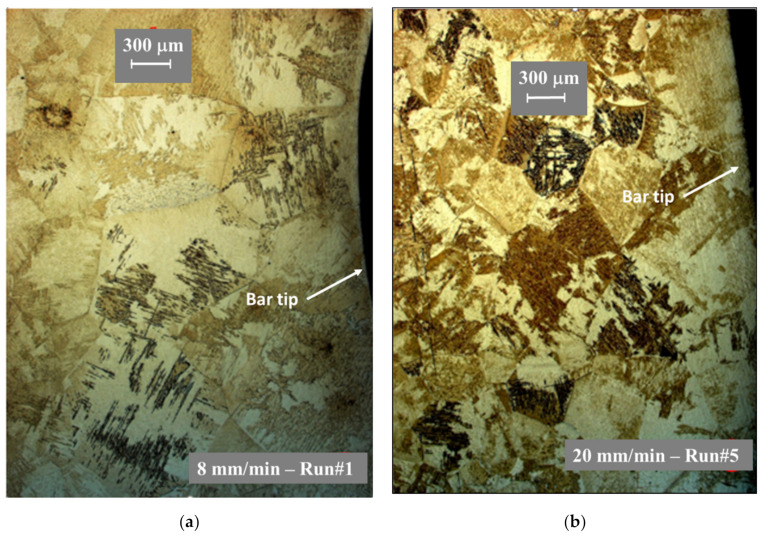
Observed grain structure near the bar tip for (**a**) low melting rate—8 mm/min and (**b**) high melting rate—20 mm/min.

Figure 6 illustrates the different grain structure in the first millimeters from the extremity of the bar. The tip of the bar is clearly distinguished on the right-hand side of each image.

The grain morphologies are equiaxed with a size noticeably larger for the lower melting rate. This is caused by the longer time this region was subjected to high temperature (above the beta transus—see below).

**Figure 7:**
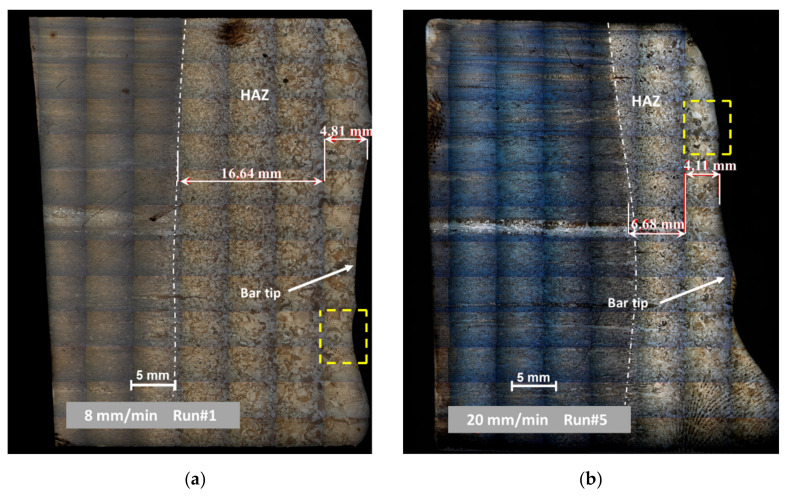
Assessment of the HAZ for (**a**) low melting rate—8 mm/min and (**b**) high melting rate—20 mm/min. Stitched optical microscope images for mapping (unit in dashed yellow).

From these local images obtained by optical microscopy, mapping pictures of the whole bar tip could be built thanks to the automatic driven system option integrated in Axiovision software (Zeiss, Iena, Germany). Indeed, this system allows to stitch, with a controlled overlap percentage, a large number of images (each square, as the ones dashed in yellow, is a single optical microscope image). These microstructure maps are shown in the Figure 7 for both trials. 

On Figure 7, the Heat Affected Zone (HAZ) can be easily unveiled because of microstructure change during heating. According to the Ti-64 phase diagram, beta grain growth occurs during heating at a temperature higher than the beta transus temperature T_αβ_ (around 880 °C). Because of the higher mobility of beta Ti for grain growth due to its single-phase solution nature whereas the alpha Ti has a lower mobility due to its multiphase nature, this heating above the transus leads to a large grain structure [18] before quenching as electron beam power is turned off. 

Consequently, HAZ boundaries have been sketched in dotted line on the images. On the left-hand side of this limit, primary Ti-64 that has not experienced the beta transus can be seen. On the other side, beta grain size enlargement can be noticed from the HAZ beginning to the bar tip. An average value for HAZ was approximately measured for two samples and determined to be around 21 and 11 mm at low and high melting rates, respectively.

### 3.4. Aluminum Depletion by Volatilization

Because of high vacuum, the main drawback of the EBM processes is a significant loss by volatilization of alloying elements exhibiting a high vapor pressure (such as aluminum in Ti-64). This provokes a pollution of the chamber walls and makes it difficult to control the chemical composition of the as-cast ingot.

The significant temperature gradient in the liquid film makes the chemical analysis of the bar tip difficult and inaccurate. This is the reason why the volatilization losses have been obtained with a chemical analysis of the cast ingots using the Glow Discharge Optical Emission Spectrometry (GDOES) technique. Table 3 reports the aluminum content of the initial bar and the mean value of the radial ingot section for the low and high melting rate runs.

The analyses emphasize that the EBDM at higher melting rate reduces the losses by volatilization. Since the overheating temperature does not change with the EB power, the volatilization flux on the bar tip remains roughly constant. It is therefore obvious that a higher melting rate reduces the residence time of the alloy in a liquid state and then the aluminum losses. This result agrees well with the literature [19,20] where the detrimental effect of low melting rate on aluminum depletion was established.

### 3.5. Results of the Numerical Simulation and Discussion

#### 3.5.1. Melting Rate and Overheating Temperature

**Figure 8:**
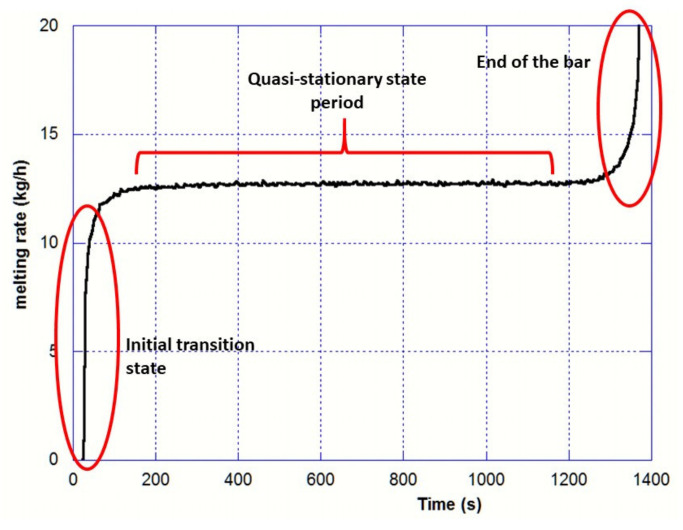
Typical shape of the melting rate (example of Run#5; 20 mm/s)—Mesh refinement (r,z): 160 × 1500.

The eight experimental runs have been simulated, and the excellent convergence of the heat transfer equation lead to a heat balance lower than 0.1%. One of the most interesting results provided by Ebmelting code is the melting rate profile as shown in Figure 8 for the Run#5 at 20 mm/min. Following the EB heating of the bar tip, the first droplets of liquid metal are formed after 30 s. The melting rate increases very steeply and then levels off at a roughly constant value corresponding to the quasi-stationary state. Finally, the melting rate rises again at the end of the consumption of the bar. The oscillation of the melting rate can be readily explained by the ablation of mesh cells, whose temperatures are greater than T_oh_. The amplitude of the oscillations is then strictly correlated to the mesh refinement.

For each experiment, a wide range of overheating temperature was set into the numerical simulation model as an input to calculate the corresponding melting rates as illustrated in Figure 8. The melting rate calculated by the model obviously decreases with the overheating temperature since a higher thermal energy is required to remove the liquid Ti-64 at the bar tip. Moreover, the heat lost by radiation ${\dot{Q}}_{\mathrm{rad}}^{\mathrm{tip}}$ increases non-linearly with T_oh_, which results in a lower melting rate. 

**Figure 9:**
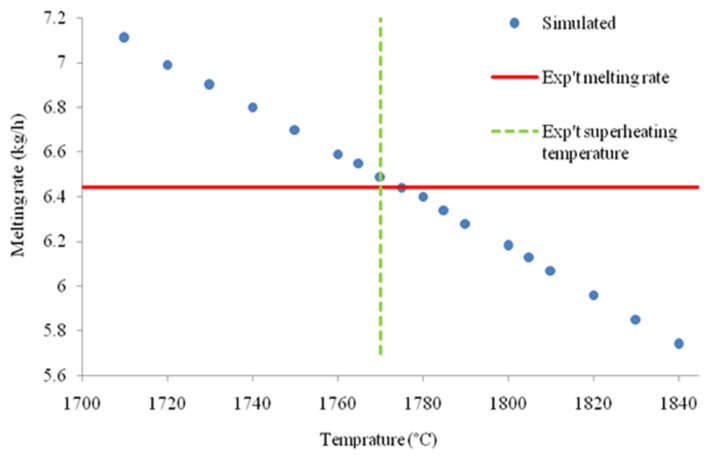
Simulated melting rate versus overheating temperature T_oh_ and measured temperature and melting rate (Run #2: 10 mm/min).

Then, the measured (continuous line) and simulated (markers) melting rates were compared as shown as an example in Figure 9. The intersection of calculated and experimental melting rate gives an assessment of the overheating temperature required in the model. 

The latter matches well with the measured tip temperature, as shown in Table 4, that gathers the simulated and experimental values for each individual run. A good agreement between measured and indirectly calculated overheating temperatures is obtained in all cases except in Run#3 where the substantial difference could be attributed to an accumulation of experimental errors.

Table 4 also confirms that the overheating temperature is not correlated with the melting rate, and its value remains in a range between 90 and 150 °C above the liquidus temperature (T_liq_ = 1670 °C). 

#### 3.5.2. Thermal Profiles and HAZ

**Figure 10:**
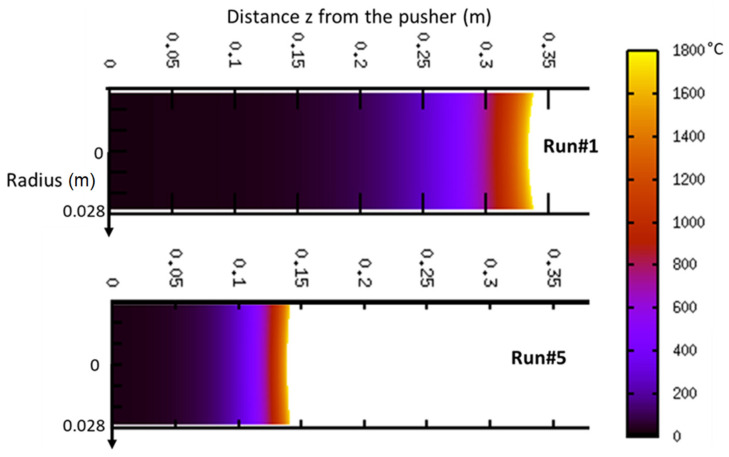
Contour of the temperature after 1000 s of melting Run#1 at 8 mm/min and Run#5 at 20 mm/min.

The numerical model provides at each time step of the simulation the temperature distribution in the bar. Figure 10 presents such computed thermal map under two different melting rates at t = 1000 s. The model predicts that the highest axial temperature gradient is confined close to the bar tip whereas the main part of the bar remains at room temperature. This thermal behavior is easily attributable to the competition between the consumption speed and the thermal diffusion velocity. Calculation of a dimensionless Péclet number allows assessment of the relative role of each of these two phenomena (ratio of advective to diffusive). It gives:

(11) $${\mathrm{Pe}}_{\mathrm{Run}1}=\frac{u_{b}\;L}{\alpha }=7.3\;\mathrm{and}\;{\mathrm{Pe}}_{\mathrm{Run}5}=27$$

where L is the bar length, u_b_ the bar speed and α the thermal diffusivity. Accordingly, these values testify that on the one hand the bar moves forward much faster than the heat diffuses and, on the other hand, the higher the melting rate, the steeper the temperature gradient.

The concave shape of the bar tip results directly from the thermal losses by radiation along the edge of the bar. This shape remains essentially constant throughout the melt, but we can notice that the curvature of the tip is more pronounced at the beginning of the melt, when EB power is low. In that case, the effect of radiation losses is proportionally more important and Ti-64 melting is, from a thermal point of view, enhanced at the center compared to the periphery. However, the discrepancy with the real shape observed in Figure 7 can be readily explained by the fact that the model does not take into account the dynamic motion of the liquid film under forces such as wetting, buoyancy and thermocapillary [21].

**Figure 11:**
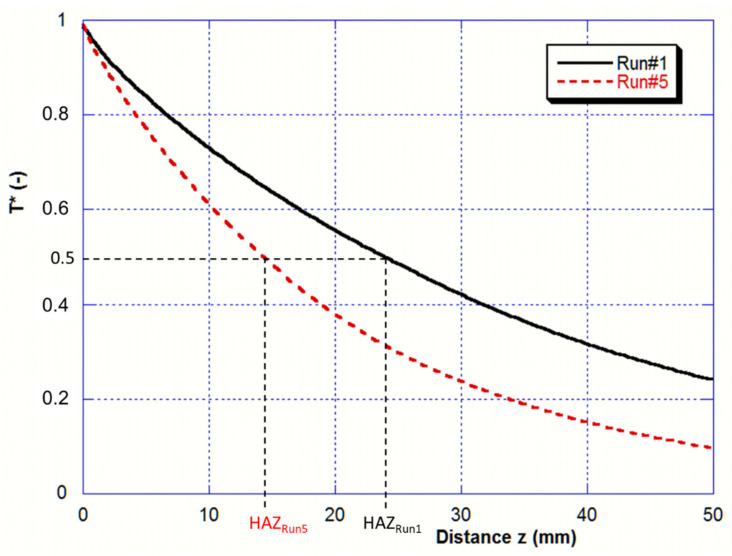
Comparison of the normalized wall temperature profiles in two cases of low and high melting rates (Run#1—8 mm/min and Run#5—20 mm/min).

As discussed above, the Heat-Affected Zone (HAZ) is the part of the bar that experiences a temperature over the beta transus T_αβ_. Profiles of normalized temperature of the bar side are drawn in Figure 11 for the two melting rates (Run#1 and Run#5) and allow determination of the predicted HAZ. The normalized temperature is given by:

(12) $$T^{*}\left(z\right)=\frac{T\left(z\right)-T_{0}}{T\left(z=0\right)-T_{0}}$$

where z is here the distance from the bar tip.

The computed HAZ values for Run#1 and Run#5 (respectively 24 and 14 mm) match quite well with the measured values (respectively 21 and 11 mm) deduced from the micrographs on Figure 7.

#### 3.5.3. Calculation of Thermal Balance

The global thermal balance applied to the bar was calculated during the quasi-stationary stage by the numerical model Ebmelting, and an example of the results is reported in Table 5. Definition of the variables are given in Section 2.2 except for the heat power requested to heat and melt the material:

(13) $${\dot{Q}}_{\mathrm{melting}}=\dot{m_{m}}\left(h_{\mathrm{liq}}\left(T_{\mathrm{oh}}\right)-h_{\mathrm{sol}}\left(T_{0}\right)\right)$$

The excellent convergence of the simulation is proved by the small balance (lower than 0.1%). The heat lost at the pusher side remains negligible as long as the bar length is much larger than the HAZ. Only during the last 100 s of the melting, ${\dot{Q}}_{\mathrm{push}}$ is no longer negligible. Finally, as a consequence of the bar consumption, it is found that the heat lost by radiation clearly decreases when the melting rate increases, as discussed in Section 3.1.

## 4. Conclusions

Experiments of melting Ti-64 bars in a laboratory electron beam furnace were compared to a numerical model accounting for the various heat transfers in the titanium bar and at its surfaces. The experiments were designed to mimic the melting step in EBDM and EBCHM processes in order to investigate the effect of operating conditions in such processes. Melting rate and beam power heating the bar tip are adjusted altogether to achieve a quasi-steady melt. After experimentally determining the emissivity of liquid titanium, the overheating temperature at the bar tip was measured for 8 sets of operating parameters. These temperature measurements supported by the numerical results provided the undisputed evidence that the overheating temperature remains roughly independent of the melting rate (and therefore the EB power applied to the bar tip) and equal to about 100 °C above the liquidus temperature. This important finding indicates that high melting rate operations favor the control of the chemical composition of the re-melted material, notably in terms of aluminum depletion by volatilization. The heat affected zone in the bar was optically determined from the grain structure after quenching for two of these sets that differ significantly (melting rate more than doubled). While the overheating temperature is constant, the HAZ was found to strongly depend on operating parameters. The predictions of the numerical model for overheating temperature and HAZ are in good agreement with the experimental measurements, which validates the hypotheses on which the model is built. 

Numerical simulations and experiments together demonstrate that the consumption speed of the raw material is much faster than the thermal diffusion velocity, and as a consequence, the higher the melting rate, the steeper the temperature gradient and the smaller the HAZ.

Overall, the experimental results on the melting of a titanium bar by EBM provide knowledge on this step that conditions industrial processes such as EBDM and EBCHM, but that was only scarcely studied in the scientific literature. The associated numerical model not only provides a way to quantify the respective contributions of several heat fluxes in the process, but since it has been validated against experiments, it also provides a relatively simple yet predictive model of EBM that can be used as input in further studies of the downstream steps that distinguish EBDM and EBCHM processes.

## Figures and Tables

**Table 1:** Thermal properties of Ti-64 used in the numerical simulations [13,14].

Properties of Ti-64	Values or Expression
ρ_l_ liquid density (kg·m^−3^)	4100
ρ_s_ solid density (kg·m^−3^)	4158
T_liq_ liquidus (°C)	1670
T_sol_ solidus (°C)	1650
L latent heat of melting (J·kg^−1^)	3.89 × 10^5^
C_p_ specific heat of solid (J·kg^−1^·K^−1^)	710
C_p_ specific heat of liquid (J·kg^−1^·K^−1^)	794
λ_s_ conductivity of solid (W·m^−1^·K^−1^)	18.4
λ_l_ conductivity of liquid (W·m^−1^·K^−1^)	22.5
ε_l_ emissivity of liquid	0.22
ε_s_ emissivity of solid	0.43 [14]

**Table 2:** Experimental values for each individual run.

Run #	Bar Feeder Speed (mm·min^−1^)	Melting Rate $\dot{m_{m}}\;(\mathrm{kg}/h)$	P_EB-tip_ (kVA)	P_EB-tot_ (kVA)	Measured Overheating Temperature (°C)
1	8	5.15	5.81	11.6	1760
2	10	6.44	6.38	12.4	1770
3	12	7.73	7.65	13.6	1762
4	15	9.66	8.33	14.8	1760
5	20	12.88	9.68	17.2	1772
6	25	15.90	12.16	21.6	1772
7	27	18.58	13.06	23.2	1784
8	30	20.65	14.00	24.8	1779

**Table 3:** Aluminum content in the raw material and in the final ingot.

	Al wt%—Initial Bar	Al wt%—Final Ingot
Run#1—8 mm/min	6.0	4.67
Run#5—20 mm/min	6.0	5.39

**Table 4:** Simulated and experimental values for each individual run.

Run #	Bar Feeder Speed (mm/min)	Melting Rate $\dot{m_{m}}\;(\mathrm{kg}/\mathrm{hr})$	P_EB-tip_ (kVA)	Experimental Overheating Temperature (°C)	Simulated Liquid Temperature T_oh_ (°C)
1	8	5.15	5.81	1760	1790
2	10	6.44	6.38	1770	1775
3	12	7.73	7.65	1762	1822
4	15	9.66	8.33	1760	1798
5	20	12.88	9.68	1772	1750
6	25	15.90	12.16	1772	1770
7	27	18.58	13.06	1784	1762
8	30	20.65	14.00	1779	1781

**Table 5:** Global thermal balance (W) of the bar calculated during quasi-stationary regime.

Watt:	$\varepsilon_{EB}P_{EB-tip}$	${\dot{Q}}_{rad}^{tip}$	${\dot{Q}}_{rad}^{side}$	${\dot{Q}}_{push}$	${\dot{Q}}_{melting}$	Balance (%)
Run#1	4068	561	964	0	2542	0.02
Run#5	6780	529	450	0	5808	0.10

## Data Availability

The data presented in this study are available on request from the corresponding author.

## References

[1] Sandell V., Hansson T., Roychowdhury S., Mansson T., Delin M., Akerfeldt P., Antti M.L. (2021). Defects in Electron Beam Melted Ti-6Al-4V: Fatigue Life Prediction Using Experimental Data and Extreme Value Statistics. Materials.

[2] Bellot J.P., Foster B., Hans S., Hess E., Ablitzer D., Mitchell A. (1997). Dissolution of hard-alpha inclusions in liquid titanium alloys. Met. Trans. B.

[3] Cen M.J., Liu Y., Chen X., Zhang H.W., Li Y.X. (2020). Calculation of flow, heat transfer and evaporation during the Electron Beam Cold Hearth Melting of Ti-6al-4V Alloy. Rare Met. Mat. Eng..

[4] Bellot J.P., Dussoubs B., Reiter G., Flinspach J. (2006). A comprehensive numerical modelling of electron beam cold hearth refining and ingot consolidation of Ti alloys. Rare Met. Mat. Eng..

[5] Vutova K., Vassileva V., Koleva E., Georgieva E., Mladenov G., Mollov D., Kardjiev M. (2010). Investigation of electron beam melting and refining of titanium and tantalum scrap. J. Mater. Process Technol..

[6] Bertram L.A., Zanner F.J. Electrode tip melting simulation during vacuum arc remelting of Inconel 718. Proceedings of the Modeling and Control of Casting and Welding Processes.

[7] Jardy A., Falk L., Ablitzer D. (1992). Energy exchanges during vacuum arc remelting. Ironmak. Steelmak..

[8] Carslaw H.S., Jaeger J.C. (1959). Conduction of Heat in Solids.

[9] Zanner F.J., Bertram L.A. Vacuum arc remelting: An overview. Proceedings of the 8th International Conference on Vacuum Metallurgy.

[10] Shiller S., Heisig U., Panzer S. (1982). Electron Beam Technology.

[11] Clites P.G., Beall R.A. (1967). A Study of Heat Transfer to Water-Cooled Copper Crucibles during Vacuum Arc Melting.

[12] Khasin G.A., Bigashev V.Z., Ermanovich N.A. (1973). Contact phenomena at bottom of an ingot in ESR and VAR processes. Steel USSR.

[13] Boivineau M., Cagran C., Doytier D., Eyraud V., Nadal M., Wilthan B., Pottlacher G. (2006). Thermo- physical properties of solid and liquid TA6V titanium alloy. Int. J. Thermophys..

[14] Shur B.A., Peletskii V.E. (2004). The Effect of Alloying Addition on the emissivity of Titanium in the Neighborhood of Polymorphous Transformation. J. High Temp..

[15] Patankar S.V. (1980). Numerical Heat Transfer and Fluid Flow.

[16] Choi W., Jourdan J., Matveichev A., Jardy A., Bellot J.P. (2017). Kinetics of Evaporation of Alloying Elements under Vacuum: Application to Ti alloys in Electron Beam Melting. High Temp. Mater. Process..

[17] Rai R., Burgardt P., Milewski J.O., Lienert T.J., DebRoy T. (2009). Heat transfer and fluid during electron beam welding of 21Cr-6Ni-9Mn steel and Ti-6Al-4V alloy. J. Phys. D Appl. Phys..

[18] Kobryn P.A., Semiatin S.L. (2003). Microstructure and texture evolution during solidification processing of Ti-6Al-4V. J. Mater. Process. Technol..

[19] Bellot J.P., Hess E., Ablitzer D. (2000). Aluminium volatilization and inclusion removal in the electron beam cold hearth melting of Ti alloys. Met. Mater. Trans. B.

[20] Westterberg K.W., McClelland M.A., Bakish R. (1994). Modeling of Material and Energy Flow in an EBCHR Casting System.

[21] Bhar R., Jourdan J., Descotes V., Jardy A. (2019). An experimental study of the inclusion behavior during maraging steel processing. Met. Res. Technol..

